# Hypoxia negates hyperglycaemia-induced chemo-resistance in breast cancer cells: the role of insulin-like growth factor binding protein 2

**DOI:** 10.18632/oncotarget.20287

**Published:** 2017-08-16

**Authors:** Athba Al Qahtani, Jeff Holly, Claire Perks

**Affiliations:** ^1^ IGFs and Metabolic Endocrinology Group, School of Clinical Sciences, University of Bristol, Learning and Research Building, Southmead Hospital, Bristol BS10 1TD, UK

**Keywords:** hyperglycaemia, hypoxia, estrogen receptor, insulin-like growth factor binding protein 2, chemo-resistance

## Abstract

**Background:**

Women who suffer from breast cancer and type II diabetes with associated hyperglycaemia respond less well to chemotherapy. We have shown that hyperglycaemia induces resistance to chemotherapy through upregulation of fatty acid synthase (FASN) in breast cancer cells and increased insulin-like binding protein 2 (IGFBP-2) in prostate cancer cells. As a tumour develops the tumour mass can outgrow the blood supply resulting in the cancer cells being exposed to hypoxia that stimulates many tumorigenic signalling pathways.

**Methods:**

We used MCF-7 and T47D breast cancer cell lines. Trypan blue dye exclusion assay was employed to assess cell death and Western immunoblotting was used to determine changes in protein abundance. Hypoxia was induced both chemically by the addition of cobalt chloride (CoCl_2_) and using a hypoxia chamber.

**Results:**

IGFBP-2 abundance increased with increasing concentrations of glucose (0-25 mM) that contributed to hyperglycaemia-induced chemo-resistance as it was abrogated by downregulating IGFBP-2 using siRNA. Production of IGFBP-2 is ER dependent: pre-treatment of MCF-7 cells with β-estradiol increased IGFBP-2 and induced chemo-resistance to doxorubicin. The hyperglycaemia-induced chemo-resistance and increases in FASN and IGFBP-2 were negated in a hypoxic environment, with levels of cell death unaffected by glucose concentrations.

**Conclusions:**

The sensitivity of breast cancer cells to chemotherapy is reduced in hyperglycaemic conditions but this effect is negated by hypoxia. These effects appear to be mediated via regulation of IGFBP-2 and FASN. Understanding the role of FASN and IGFBP-2 in chemo-resistance could provide a novel target for improving the effectiveness of breast cancer treatment.

## INTRODUCTION

Cancer is complex and its initiation and progression involves many factors including genetic and environmental influences that include lifestyle. Accumulating epidemiological studies have suggested that there is a positive association between obesity, hypernutrition and increased risk of cancer. Over the past two decades, an estimation of 14% of all cancer mortality in men and 20% in woman are attributed to obesity and overweight, respectively [1]. In recent decades, increased caloric intake and reduced energy expenditure have increasingly become the general lifestyle in many different countries [2]. Adipose tissue in obesity exhibits significant changes in hormone secretion, adipokine, and cytokine production. Obesity is also associated with insulin resistance, type 2 diabetes, atherosclerosis and hyperglycaemia. High concentrations of insulin and glucose promote growth and survival of malignant cells [3]. Cancer cells favour high glucose concentrations and glycolytic metabolism, which provide tumour cells with intrinsic growth stimulation and anti-apoptotic mechanisms under the influence of the Warburg effect [4].

IGFBP-2 is an essential regulator of the IGF axis. IGFBP-2 is one of the most abundant IGFBPs and its importance lies, at least in part, in its ability to modulate the bioavailability of IGF-I and –II [5]. IGFBP-2 can however also act independently of IGFs and there is evidence suggesting that IGFBP-2 activates intrinsic signalling cascades through its Arg-Gly-Asp (RGD) motif and the interaction with integrin receptors, in particular the α5β1 integrin [6]. The expression of IGFBP-2 is positively associated with tumour progression in various types of cancers. Elevated serum levels of IGFBP-2 and abundance of IGFBP-2 in solid tumours has been reported in a variety of malignancies including breast [7], prostate [8], lung [9], and colon [10]. Other studies have shown that glucose metabolism greatly influences the expression of IGFBP-2. This was shown in prostate cancer cells where IGFBP-2 transcription was induced in high glucose due to acetylation of histones H3 and H4 associated with the *Igfbp-2* promoter region. This upregulation also influenced the ability of the cancer cells to respond to treatments and induced resistance to chemotherapy in hyperglycaemic conditions [11].

Glucose serves as a substrate for fatty acid synthase (FASN) and FASN is highly expressed in malignant cells including breast cancer [12]. We and others have shown that silencing FASN using siRNA leads to improved sensitivity of breast cancer cells to chemotherapy [13, 14]. Our previous studies have shown that the expression of FASN is higher in malignant cells compared to non-malignant cells [14]. In addition, inhibition of FASN eliminates the survival effects conferred by high glucose against cell death induced by both paclitaxel and 5-fluorouracil in MCF-7 and T47D breast cancer cells indicating that FASN might be a mediator in the pathway of resistance to chemotherapies in a hyperglycaemic environment.

Numerous studies have reported that hypoxia contributes to many human diseases. The effect of hypoxia is chiefly mediated by the transcription factor hypoxia inducible factor-I (HIF-I). HIF-I is composed of two subunits, HIF-Iα and HIF-Iβ. The stability of HIF-Iα determines its transcriptional activity. Under hypoxia, HIF-Iα is stabilised and translocated to the nucleus where it regulates the expression of many genes [15]. Hypoxia influences many biological functions including cell proliferation, angiogenesis, apoptosis and insulin resistance [16, 17]. Accumulating evidence suggests that adipose tissue in obese individuals receives an inadequate supply of oxygen, leading to the induction of HIF-Iα [18, 19]. Moreover, the blood flow in subcutaneous adipose tissue is lowered in obese people compared to lean people [20]. Also, the natural rapid diet-induced increase in subcutaneous blood flow [21] is significantly reduced or even diminished in obese individuals [20]. All these factors contribute to a potentially higher exposure to hypoxia.

Obesity is associated with various metabolic dysfunctions including insulin resistance, type 2 diabetes, and elevated levels of glucose in the blood [3]. Obesity initiates insulin resistance, which triggers two cancer-promoting factors; hyperinsulinemia and hyperglycaemia. Sustained insulin resistance leads to hyperglycaemia that subjects cells including tumour cells to abnormally high concentrations of glucose. High levels of insulin and glucose positively drive cancer cell growth and survival. That is because tumour cells favour glycolytic metabolism [4], utilising high glucose as a fuel for survival. In this study, we aimed to assess the impact of hypoxia on the sensitivity of breast cancer cells to chemotherapy and its effect on hyperglycaemia-induced chemo-resistance and the underlying mechanisms.

## RESULTS

### Glucose increases IGFBP-2 at the protein and mRNA levels

To investigate the effect of glucose on the abundance of IGFBP-2, breast cancer cells MCF-7 and T47D were exposed to different levels of glucose (5mM, 9mM and 25mM) and the levels of IGFBP-2 secreted into the media and within the cells was examined by Western immunoblotting.

**Figure 1:**
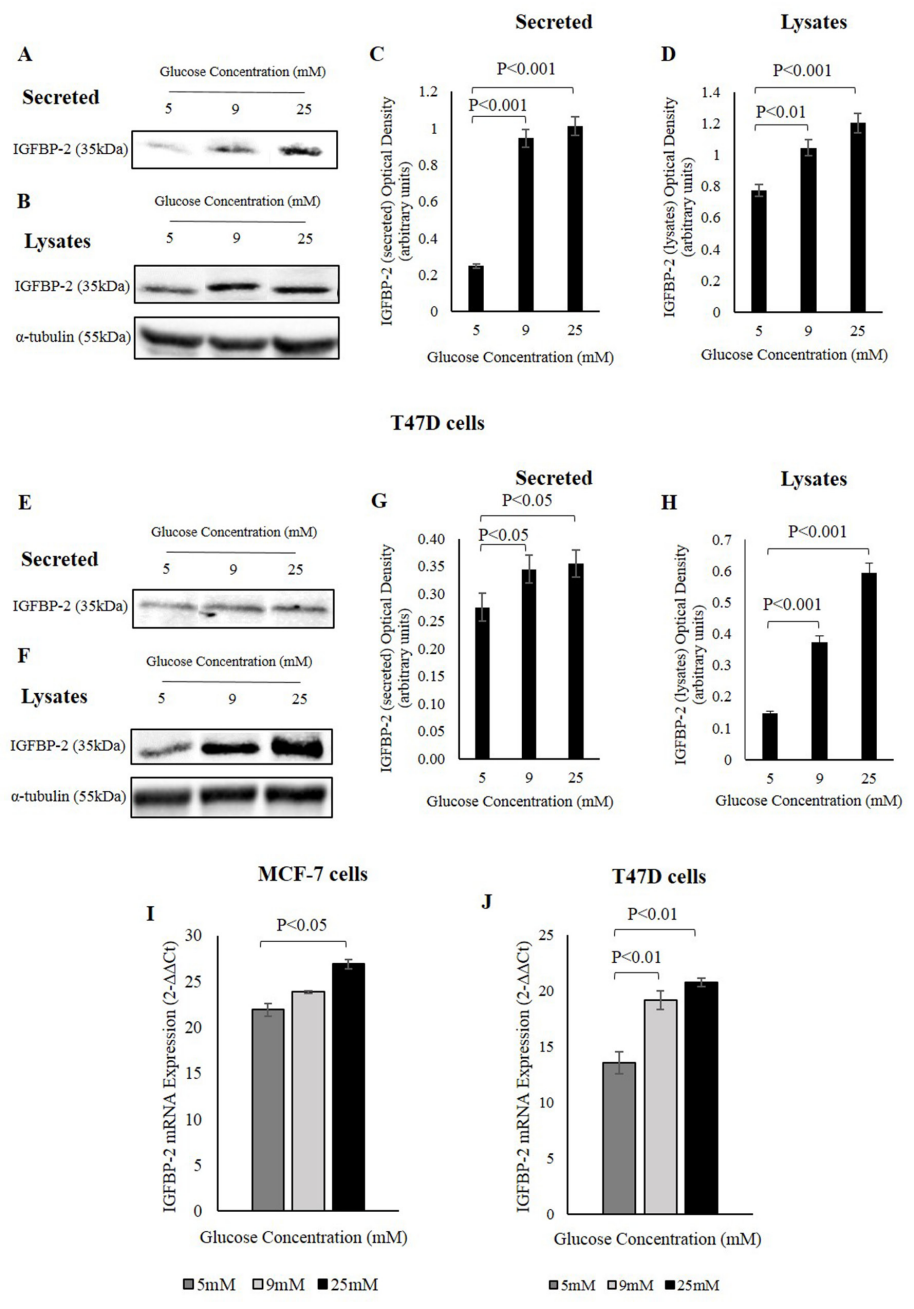
IGFBP-2 increases in response to different glucose levels MCF-7 cells were treated with 5, 9, and 25mM glucose for 48h and the abundance of secreted **(A)** and endogenous **(B)** IGFBP-2 was examined by Western immunoblotting. The optical densitometry measurements of IGFBP-2 secreted **(C)** and in lysates **(D)** in MCF-7 cells. T47D cells were treated with 5, 9, and 25mM glucose for 48h and the abundance of secreted **(E)** and in lysates **(F)** IGFBP-2 was examined by Western immunoblotting. The optical densitometry measurements of IGFBP-2 secreted **(G)** and in lysates **(H)** in T47D cells. **(I)** MCF-7 cells were seeded at 0.4x10^6^ cells in 5mM glucose growth medium for 24h prior to switching to SFM of different glucose concentrations 5, 9, and 25mM glucose for 24h. The cells were lysed with Trizol and the level of IGFBP-2 mRNA expression was determined by real-time PCR. **(J)** T47D cells were treated the same way as MCF-7 cells in 5, 9, and 25mM glucose for 24h, and the level of IGFBP-2 expression was determined by real-time PCR. 18S was used a internal housekeeping gene. The graphs represent the mean±SEM of three independent repeats each conducted in triplicate.

MCF-7 cells exhibited an approximate 4-fold increase in secreted IGFBP-2 levels at 9mM and 25mM glucose compared to 5mM glucose (Figure 1A and 1C). Cellular IGFBP-2 also increased approximately 1.5 fold at 25mM glucose compared to 5mM glucose (Figure 1B and 1D). T47D cells showed a similar pattern to MCF-7 cells in that IGFBP-2 abundance increased in response to glucose. At 9mM and 25mM glucose, secreted IGFBP-2 showed an approximate one-fold increase at 9mM and 25mM glucose compared to 5mM glucose (Figure 1E and 1G). Endogenous IGFBP-2 also increased at 9mM and 25mM glucose by approximately 2.5 and 4 fold, respectively, compared to 5mM glucose (Figure 1F and 1H).

To determine whether the glucose-induced secretion was regulated at the transcriptional level in addition to the protein level, the mRNA expression of IGFBP-2 in MCF-7 and T47D cells was determined using real-time PCR. Glucose increased IGFBP-2 expression by approximately 1.2 fold (P<0.05) in 25mM glucose compared to 5mM glucose in MCF-7 cells (Figure 1I), and a greater response was observed in T47D cells where IGFBP-2 expression increased approximately 1.4 fold (P<0.01) in 9mM glucose and 1.5 fold (P<0.01) in 25mM glucose (Figure 1J).

Previously, we reported that in prostate cancer cells glucose increased resistance to chemotherapy through upregulating IGFBP-2 [11]. Increased acetylation of histones associated with the *Igfbp-2* gene promoter in hyperglycaemic conditions was associated with increased IGFBP-2 transcription and induced chemo-resistance [11]. This glucose mediated regulation of IGFBP-2, however, was different in breast cancer cells. The histones associated with *Igfbp-2* gene promoter were not acetylated or de-methylated by high glucose suggesting that the hyperglycaemic induction of IGFBP-2 was via alternative mechanisms (data not shown).

### Does the glucose-induced increase in IGFBP-2 contribute to hyperglycaemia-induced chemo-resistance?

Having determined that glucose increased the levels of IGFBP-2 both at the protein and mRNA levels in breast cancer cells, the role of IGFBP-2 in the response of breast cancer cells to chemotherapy was investigated.

**Figure 2:**
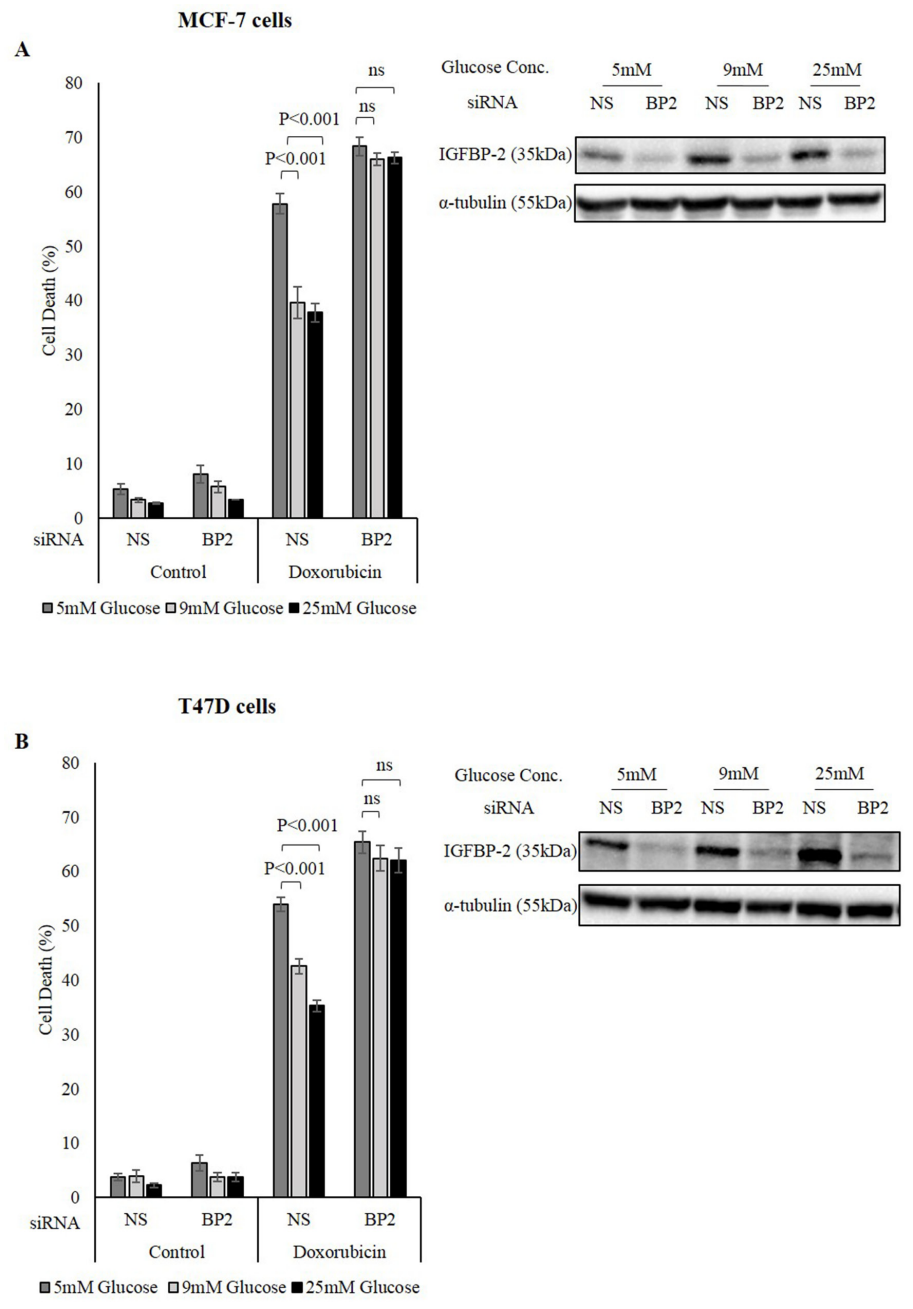
The role of IGFBP-2 in hyperglycaemia-induced chemo-resistance MCF-7 cells **(A)** and T47D cells **(B)** were incubated in different glucose conditions (5mM, 9mM and 25mM) and treated in the presence and absence of IGFBP-2 siRNA. The cells were treated with 1μM doxorubicin. Cell death was measured by a trypan blue dye exclusion assay. Inserts: IGFBP-2 abundance in 5, 9, 25mM glucose in the presence and absence of IGFBP-2 siRNA where the total cell extract was analysed by Western immunoblotting. The graph represents the mean±SEM of three independent repeats each conducted in triplicate; NS= non-silencing siRNA, BP2= IGFBP-2 siRNA.

MCF-7 cells were transfected with IGFBP-2 siRNA to silence its expression and then were grown in different glucose concentrations (5mM, 9mM and 25mM glucose) prior to treatment with doxorubicin. MCF-7 cells acquired resistance to doxorubicin in 9mM and 25mM glucose and the level of cell death decreased from 60% at 5mM glucose to 39.6% at 9mM glucose (P<0.01) and 37.7% at 25mM glucose (P<0.01) for MCF-7 cells. However, this glucose-induced resistance to chemotherapy was negated when IGFBP-2 was silenced. Sensitivity to doxorubicin was restored in MCF-7 cells and the level of cell death was similar in all levels of glucose at 66% cell death in 9mM and 25mM glucose compared to 68% in 5mM glucose (Figure 2A). The transfection with IGFBP-2 siRNA was successful in suppressing IGFBP-2 production as indicated in the insert of Figure 2. These data suggest that IGFBP-2 plays a significant role in how the breast cancer cells respond to chemotherapy in a hyperglycemic environment. These findings were further confirmed in T47D cells where the hyperglycemia-induced chemo-resistance was also negated when IGFBP-2 expression was silenced (Figure 2B).

### Investigation of the response of MCF-7 cells to chemotherapy following addition of recombinant IGFBP-2

Having shown that IGFBP-2 plays a role in hyperglycaemia-induced chemo-resistance, adding exogenous recombinant IGFBP-2 to breast cancer cells and monitoring their response to chemotherapy was performed to further investigate the effect of IGFBP-2 on survival.

**Figure 3:**
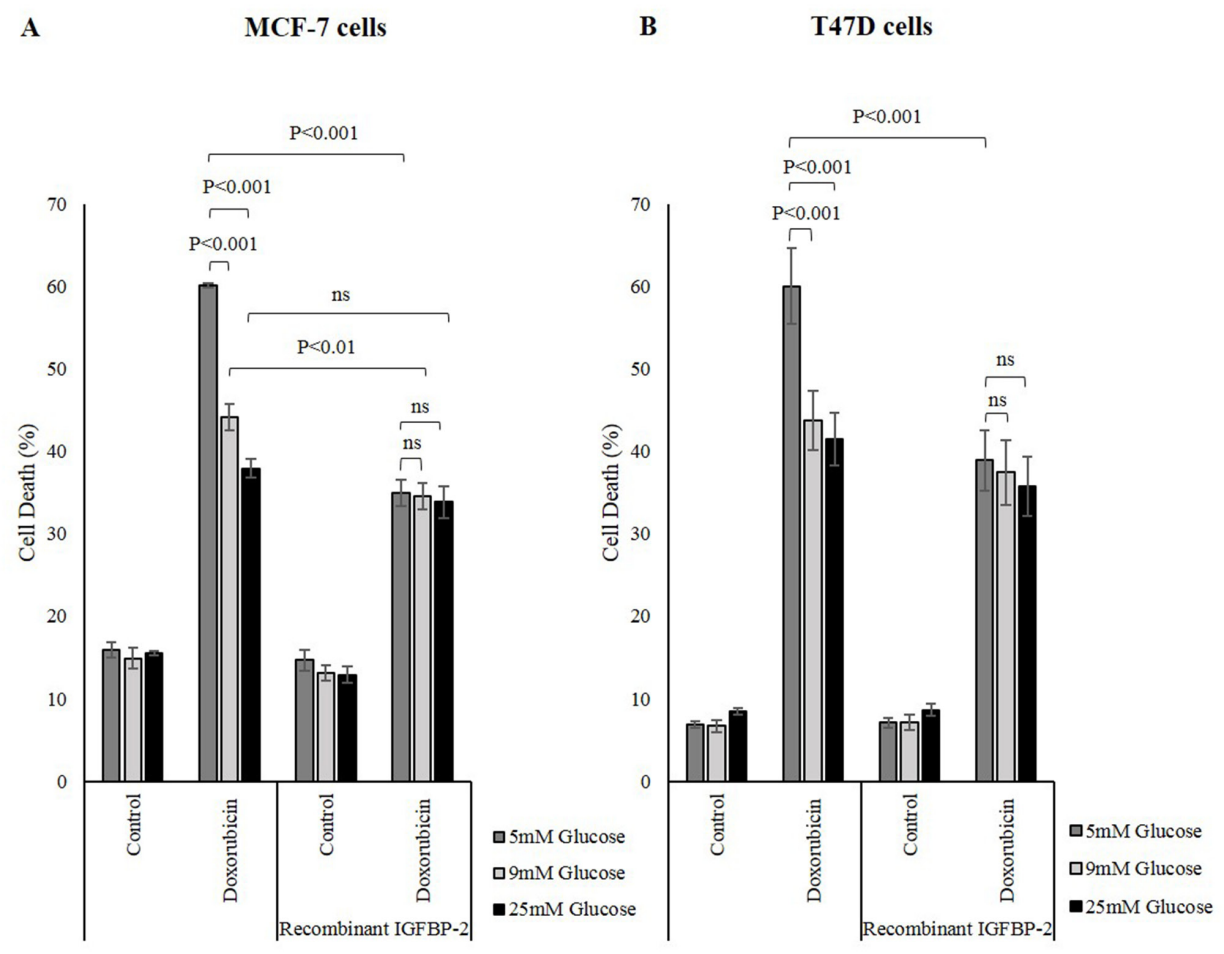
Human recombinant IGFBP-2 promotes survival against chemotherapy in breast cancer cells **(A)** MCF-7 cells were seed at 0.1x10^6^ cells then were treated with 100ng/ml human recombinant IGFBP-2 in 5mM, 9mM and 25mM glucose. The cells were exposed to 1μM doxorubicin for 24h and cell death was measured by a cell count and viability assay. **(B)** T47D cells were seed at 0.1x10^6^ cells then were treated with 200ng/ml human recombinant IGFBP-2 in 5mM, 9mM and 25mM glucose. The cells were exposed to 5μM doxorubicin for 24h and cell death was measured by a cell count and viability assay. The graphs represent the mean±SEM of three independent repeats each conducted in triplicate.

Glucose exerted a protective effect on cell survival as treating MCF-7 cells with doxorubicin induced more cell death in 5mM glucose, 60.1% compared to 44.2% in 9mM and 37.9% in 25mM glucose. The level of cell death was reduced further in all three glucose concentrations to approximately 34.6% when the cells where treated with exogenous human recombinant IGFBP-2 (Figure 3A). Similar to MCF-7 cells, T47D cells were more resistant to doxorubicin in 9mM and 25mM glucose compared to 5mM glucose. The level of cell death was 60.0% in 5mM glucose and much lower at 43.8% in 9mM and 41.5% in 25mM glucose. This doxorubicin-induced cell death was decreased when the cells were treated with exogenous IGFBP-2. The level of cell death dropped significantly in 5mM glucose from 60.0% to 38.9% upon IGFBP-2 treatment. The levels of cell death in 9mM and 25mM glucose were not significantly different at 37.4% and 35.7%, respectively (Figure 3B). This confirms that IGFBP-2 is protective against doxorubicin-induced cell death. This is consistent with the data that shows the involvement of IGFBP-2 in altered sensitivity of cancer cells to chemotherapy when IGFBP-2 expression is silenced. Having shown previously that IGFBP-2 is a negative regulator of PTEN that leads to increased Akt activation (see Supplementary Figure 2) [22], we also show that high glucose is associated with an anticipated increase in p-Akt consistent with our previous data. (Supplementary Figure 1).

### Estrogen-induced IGFBP-2 secretion is regulated by nuclear ERs but not membrane ERs

Estrogen receptors (ERs) are an essential group of receptors, which can vary in the protein conformation, location and function. There are two forms of ERs, nuclear ERs, members of the intracellular receptor family, mainly consisting of ERα and ERβ, and membrane ERs some of which belong to G-protein coupled receptors [23]. In this study, two forms of 17β-estradiol were used: human 17β-estradiol and 17β-estradiol conjugated with BSA. The former binds to all ERs whereas the conjugated 17β-estradiol with BSA is not internalized by cells and can only bind to ER found on the cell surface, i.e. membrane ERs. The estrogen receptor is a regulator of IGFBP-2 [22], and it is well known that 17β-estradiol via the nuclear ERα regulates IGFBP-2 but we wished to assess if there was also a contribution from 17β-estradiol activity via membrane receptors.

**Figure 4:**
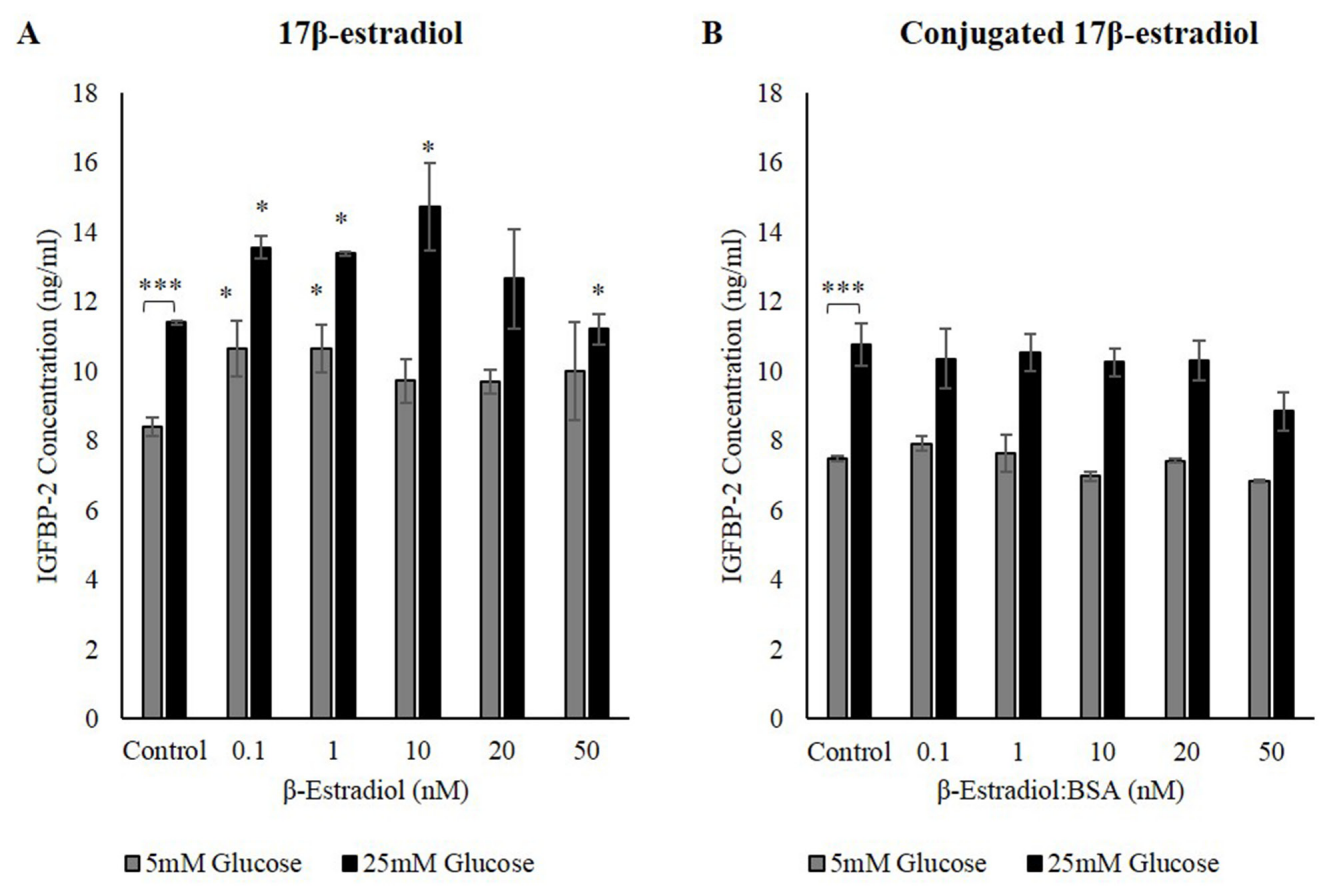
The effect sensitizing ERs on IGFBP-2 secretion MCF-7 cells were seeded at 0.0125x10^6^ cells then were treated with various doses of 17β-estradiol and conjugated 17β-estradiol ranging from 0nM to 50nM for 24h. IGFBP-2 concentrations were determined by ELISA after treatment with 17β-estradiol **(A)** and conjugated 17β-estradiol **(B)** The graphs represents the mean±SEM of three independent repeats each conducted in triplicate (* P<0.05, *** P<0.001).

Treatment with 17β-estradiol induced IGFBP-2 secretion in 5mM glucose from 8.4ng/ml to its highest 10.6ng/ml at 0.1nM and 1nM 17β-estradiol. Similarly in 25mM glucose IGFBP-2 concentrations increased from 11.4ng/ml to approximately 13.6ng/ml at 0.1nM and 1nM 17β-estradiol, and up to 14.7ng/ml at 10nM 17β-estradiol (Figure 4A). Treating the cells with the conjugated form of 17β-estradiol had no effect on IGFBP-2 secretion (Figure 4B). These data suggest that the estrogen-induced increase in IGFBP-2 is probably controlled mainly through nuclear estrogen receptors and not via membrane estrogen receptors.

### Nuclear ERs increase IGFBP-2 secretion that is associated with an increase in FASN and is protective against chemotherapy

**Figure 5:**
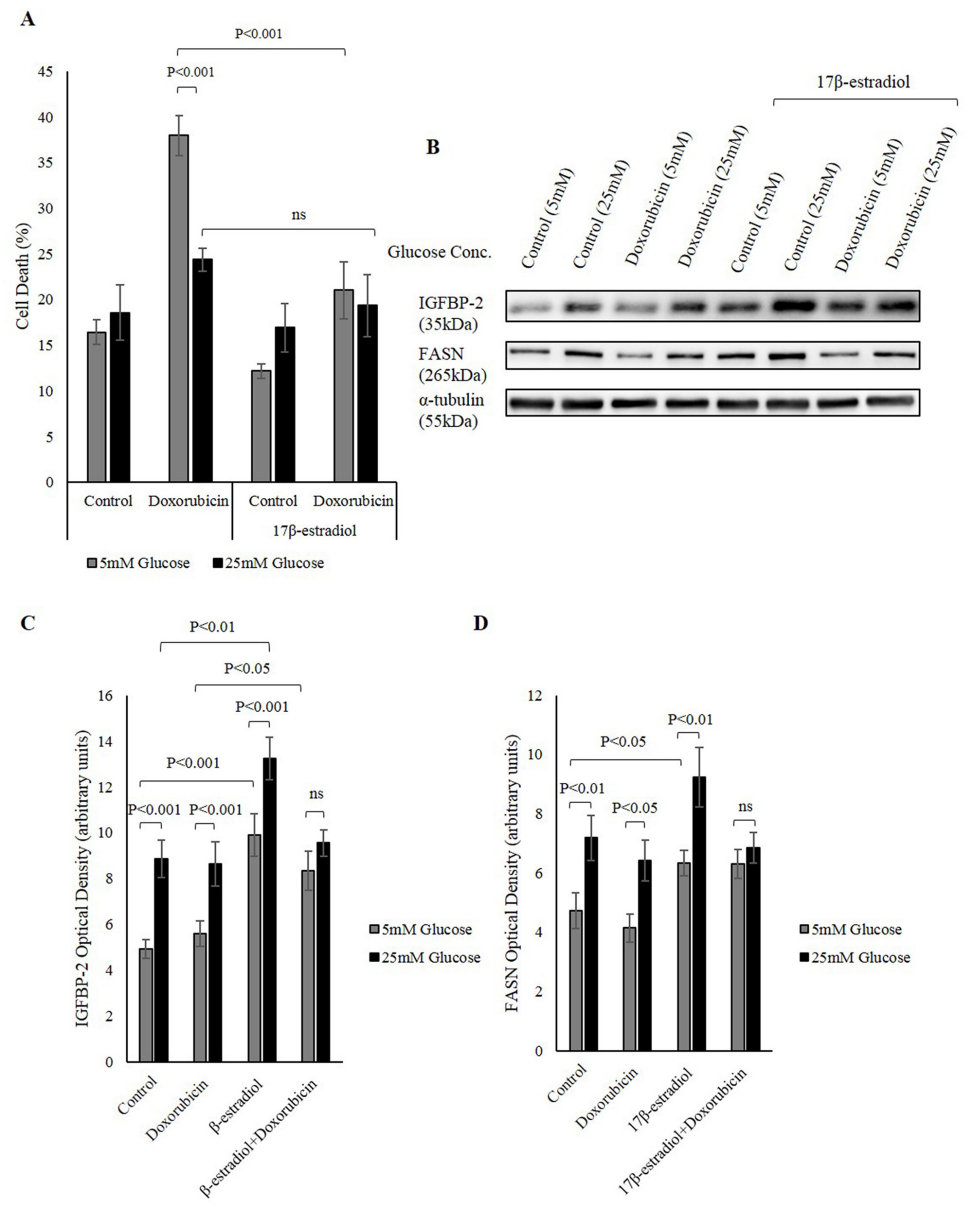
Nuclear ERs increase IGFBP-2 levels that is protective against chemotherapy **(A)** MCF-7 cells were seeded at 0.1x10^6^ cells in 5mM growth medium then were serum starved for 24h in 5mM and 25mM glucose SFM. The cells were treated with 17β-estradiol for 48h and doxorubicin was introduced in the last 24h of incubation. Trypan blue exclusion assay was used to determine the level of cell death. **(B)** Western immunoblotting showing changes in IGFBP-2 and FASN protein abundance in 5mM and 25mM glucose in the presence and absence of 17β-estradiol. Optical densitometry measurements of IGFBP-2 **(C)** and FASN **(D)** in response to 17β-estradiol in 5mM and 25mM glucose. The graphs represent the mean±SEM of three independent experiments each performed in triplicate.

Having shown that IGFBP-2 is involved in hyperglycaemia-induced chemo-resistance and that IGFBP-2 secretion was also induced by activation of nuclear ERs, we then investigated the effect of inducing IGFBP-2 secretion in breast cancer cells by estrogen and then monitoring their response to chemotherapy. MCF-7 cells were treated with 1nM 17β-estradiol in 5mM and 25mM glucose then dosed with doxorubicin. Treating MCF-7 cells with 17β-estradiol enhanced cell survival in response to doxorubicin, which was associated with an increase in IGFBP-2 secretion induced by 17β-estradiol. The levels of cell death in 5mM glucose was reduced to a level similar to the level of cell death in 25mM glucose under 17β-estradiol treatment (Figure 5A and 5B). This would be consistent with IGFBP-2 playing a protective role in inducing resistance to chemotherapy in hyperglycaemic conditions.

We have previously shown that FASN contributes to hyperglycaemia-induced chemo-resistance [14]. We have also shown that the increase in FASN in hyperglycaemic conditions was associated with an increase in the phosphorylation and nuclear translocation of ER via the MAPK pathway [24]. In this study we have examined whether this increase in IGFBP-2 through nuclear ERs involves the FASN pathway. Consistent with our previous data, FASN abundance increased in 25mM glucose compared to 5mM glucose, and this increase was further enhanced under 17β–estradiol treatment. This increase in FASN after 17β–estradiol treatment might contribute to the reduction seen in cell death induced by doxorubicin (Figure 5B, 5C and 5D).

### Hypoxia negates the hyperglycaemia-induced chemo-resistance

As the tumour develops, the tumor mass can frequently outgrow the blood supply resulting in the cells being exposed to hypoxia. Here, we investigated the effect of hypoxia on IGFBP-2 in breast cancer cells and how this affected chemo-sensitivity in euglycaemic and hyperglycaemic conditions.

**Figure 6:**
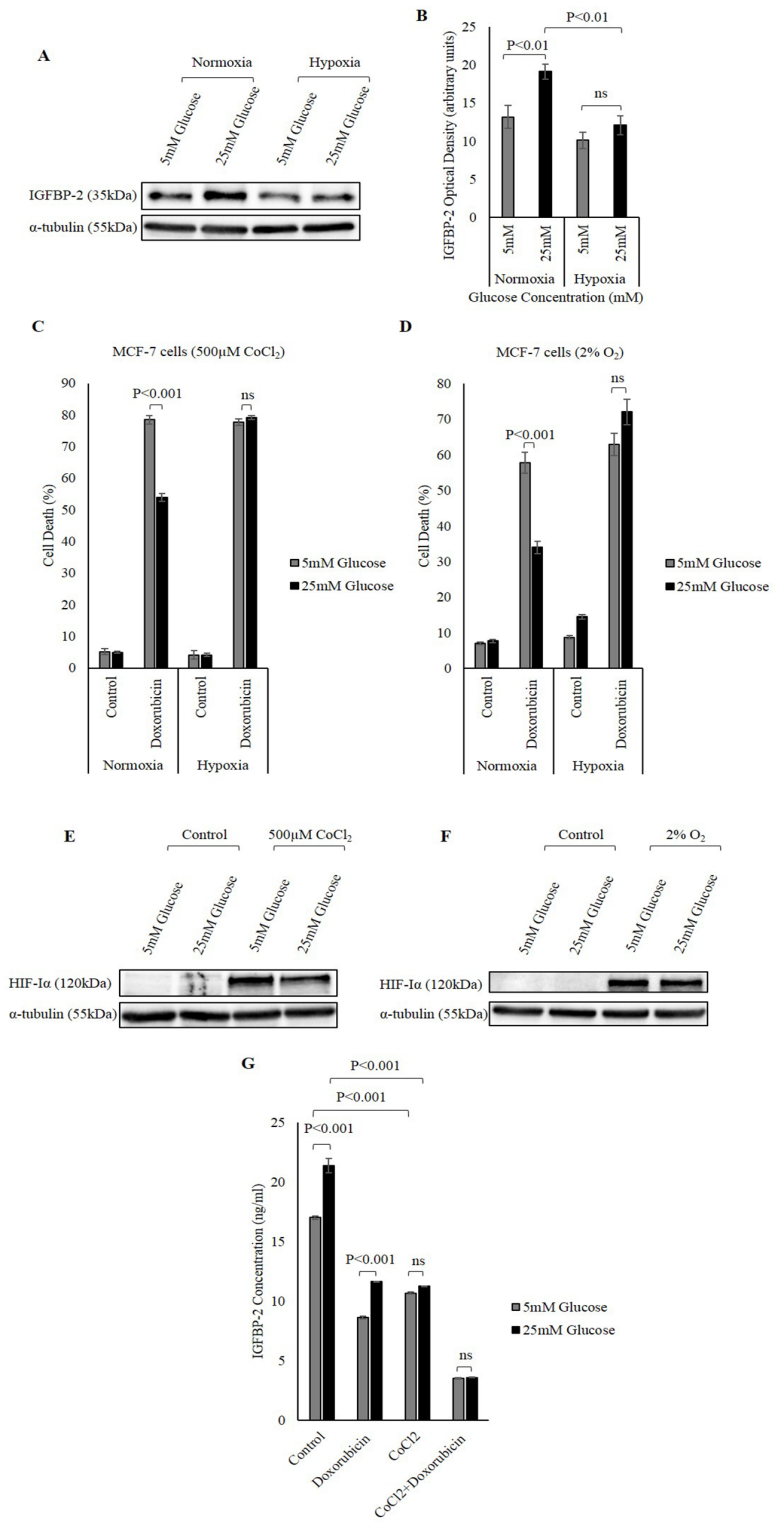
The effect of hypoxia on hyperglycaemia-induced chemo-resistance **(A)** MCF-7 cells were treated with 500μM CoCl_2_. Changes in the abundance of IGFBP-2 was analysed using Western immunoblotting. **(B)** Densitometry measurements of IGFBP-2 abundance in normoxic and hypoxic conditions in MCF-7 cells treated in 5mM and 25mM glucose. Hypoxia was chemically-induced in MCF-7 cells by 500μM CoCl_2_ **(C)** and b low levels of oxygen (2% O_2_) **(D)** in 5mM and 25mM glucose and the cells were treated with 1μM doxorubicin for 24h. The rate of cell death was analysed using a trypan blue dye exclusion assay. HIF-Iα was used as an indicator of successful induction of hypoxia by 500μM CoCl_2_ **(E)** and 2% O_2_ **(F)** α-tubulin was used as a loading control. **(G)** Changes in IGFBP-2 concentrations in MCF-7 cells treated in 5mM and 25mM glucose in the presence or absence of 500μM CoCl_2_ and 1μM doxorubicin determined using ELISA. The graph represents the mean±SEM of three independent repeats each conducted in triplicate.

Exposing MCF-7 cells to hypoxia reduced the abundance of IGFBP-2. Interestingly, the glucose-induced increase in IGFBP-2 was also negated under hypoxia induced by CoCl_2_ (Figure 6A and 6B).

Having established that hypoxia reduced the abundance of IGFBP-2, we further investigated the effect of this hypoxia-induced reduction in IGFBP-2 on the response of breast cancer cells to chemotherapy. MCF-7 cells were exposed to hypoxia, using either CoCl_2_ or grown in a hypoxic chamber with 2% O_2_, in the presence and absence of doxorubicin in different glucose conditions (5mM, 9mM and 25mM glucose). In euglycaemic conditions the amount of cell death induced by doxorubicin was not affected by hypoxia.

Exposing MCF-7 cells to 25mM glucose in normal oxygenated conditions induced resistance to doxorubicin where levels of cell death were reduced from 78.5% in 5mM glucose to 53.8% in 25mM glucose. Hypoxia induced by CoCl_2_ then negated this effect of high glucose on the response of MCF-7 cells to doxorubicin. The death induced by doxorubicin under hypoxia was not significantly different in both levels of glucose where cell death was 77.8% in 5mM glucose and 79.1% in 25mM glucose (Figure 6C). Similarly, incubating MCF-7 cells in 2% O_2_ negated the effect of hyperglycemia-induced chemo-resistance and increased cell death in 25mM glucose from 33.9% to 72.1% (Figure 6D). HIF-Iα was indicative of successful induction of hypoxia (Figure 6E and 6F). Similar findings were observed in T47D cells (data not shown). This increase in cell death under hypoxia in high glucose was associated with a reduction in IGFBP-2 (Figure 6G), consistent with IGFBP-2 playing a role in hypoxia negating the effect of glucose-induced resistance to chemotherapy.

### Hypoxia also down-regulates FASN, ERα and Akt activation

Previous studies in our laboratory have shown that IGFBP-2 is a novel regulator of the ERα, and that FASN and ERα overexpression in hyperglycaemic conditions contributes to chemo-resistance in breast cancer cells. IGFBP-2 has long been known to modulate IGF and integrin signalling [25]. PI3K/Akt signalling pathway plays a fundamental role in IGFBP-2 stimulation in breast cancer [26]. Here, we investigated whether hypoxia impacted on FASN, ERα and Akt activation that we previously identified as important players (Supplementary Figure 2).

**Figure 7:**
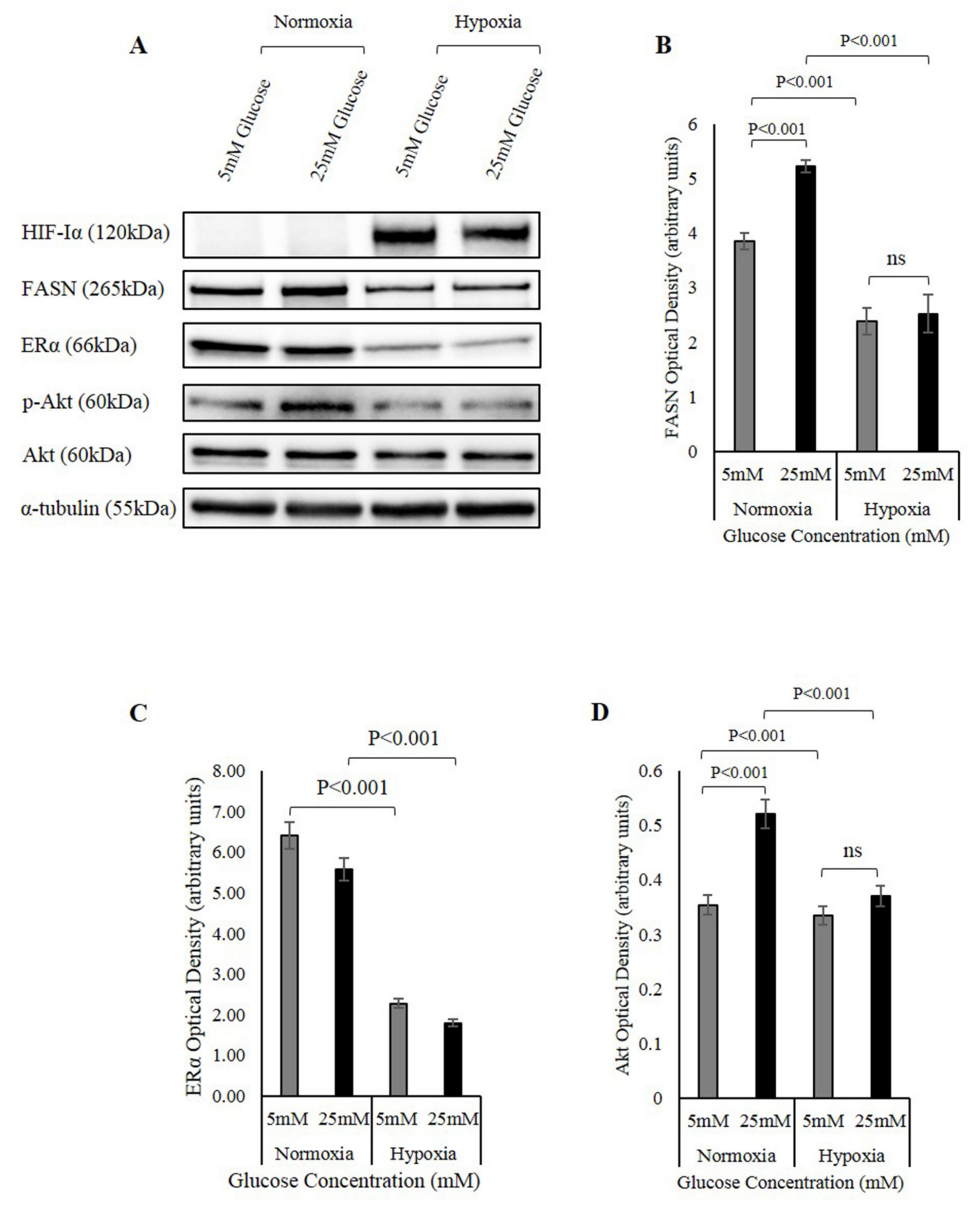
The involvement of the FASN/ERα/Akt pathway in hypoxia regulation of IGFBP-2 **(A)** MCF-7 cells were seeded at 0.1x10^6^ cells in 5mM growth medium. The cells were then treated in 5mM and 25mM glucose and were exposed to hypoxia. Changes in the abundance of HIF-Iα, FASN, ERα, Akt and p-Akt were analysed using Western immunoblotting. Densitometry measurements of changes in the abundance of FASN **(B)** ER **(C)** p-Akt/Akt **(D)** under normal and hypoxic conditions in 5mM and 25mM glucose. The graphs represent the mean±SEM of three independent repeats each conducted in triplicate.

As before, high 25mM glucose increased the abundance of FASN, however this increase was negated when the cells were exposed to hypoxia. FASN levels at both 5mM and 25mM glucose were reduced and the glucose-induced increase in FASN was completely negated (Figure 7A and 7B). Moreover, hypoxia also reduced the abundance of ERα in MCF-7 cells (Figure 7A and 7C). In addition, Akt activation increased in 25mM glucose, and this is consistent with the increase of IGFBP-2 seen in hyperglycaemia. The activity however was significantly reduced upon exposure to hypoxia. Hypoxia reduced the activity of Akt at both glucose levels and the glucose induced increase observed in 25mM glucose was completely negated (Figure 7A and 7D).

## DISCUSSION

Hyperglycaemia is associated with metabolic imbalance and type 2 diabetes and has also been linked to a high risk of breast cancers. Patients who suffer from obesity and diabetes respond less well to chemotherapy [27]. Our previous study has shown that treating breast cancer cells with doxorubicin was less effective in high glucose compared to normal glucose levels [14]. These data were consistent with epidemiological studies where breast cancer patients with diabetes have poorer responsiveness to treatment [28]. In this study we used a range of glucose concentrations to represent euglycaemic (5mM), those of a well-controlled diabetic (9mM) and physiologically extreme (25mM) levels of glucose. Resistance to doxorubicin-induced apoptosis that correlated with an increase in IGFBP-2 abundance and secretion was observed at both high levels of glucose used in this study. This study assesses extremes of hypoxia and levels of glucose and presumably in different parts of the tumour the interaction between levels of glucose and hypoxia will be graded. Understanding the balance between hypoxia and hyperglycaemia during malignant transformation is important in identifying potential mechanistic targets/biomarkers: one of which could be IGFBP-2.

Studies conducted on oesophageal adenocarcinoma cells have also indicated that IGFBP-2 modulates the response of cells to chemotherapy [29]. In primary oesophageal carcinoma patients, increased expression of IGFBP-2 correlated with shorter disease-free lifespan and induced resistance to chemotherapy. Silencing the expression of IGFBP-2 restored the sensitivity of oesophageal carcinoma cells to cisplatin [29]. Modulation of IGFBP-2 expression was shown to increase proliferation, migration, and invasion suggesting that IGFBP-2 has metastatic potential. The study also highlighted that Akt and MAPK signalling pathways were implicated in the chemo-sensitization. IGFBP-2 stimulated activation of PI3K and MAPK by both IGF-dependent and independent mechanisms [29]. This study on oesophageal carcinoma cells was consistent with our studies on breast cancer cells. We have previously shown that hyperglycaemia-induced chemo-resistance is dependent on FASN and ERα phosphorylation and translocation to the nucleus. The phosphorylation of ERα was mediated by MAPK in high glucose [24]. Here, we have shown that estrogen increased the abundance of IGFBP-2 through nuclear ERα with little contribution from the membrane estrogen receptors. Silencing the expression of IGFBP-2 using siRNA negated the hyperglycaemia-induced chemo-resistance and restored the sensitivity of breast cancer cells to doxorubicin in high glucose.

Studies have suggested that IGFBP-2 has metabolic effects. Increased expression of IGFBP-2 has been correlated with poor blood glucose clearance. IGFBP-2 harbours an integrin-binding domain Arg-Gly-Asp (RGD) that is involved in IGF-I independent effects [30]. This study examined whether or not high IGFBP-2 levels with an RGD motif would block glucose clearance after glucose oral administration. Transgenic mice that over-expressed IGFBP-2 showed impaired glucose handling after an oral glucose tolerance test but mice expressing a mutant IGFBP-2 carrying an Arg-Gly-Glu (RGE) instead of an RGD motif showed no such deterioration in glucose handling. Moreover, lower levels of the glucose transporter proteins (Glut-4) were expressed on the plasma membrane of muscle tissue isolated from the transgenic mice after glucose administration. The low levels of Glut-4 were associated with impaired glucose tolerance. This study identified that elevated integrin-linked kinase and focal adhesion kinase levels were dependent on the existence of the RGD motif. The RGD motif was important for glucose clearance in IGFBP-2 transgenic mice and that Glut-4 expression was regulated by IGFBP-2 in an RGD-dependent manner [30]. In this study we have shown that glucose-induced IGFBP-2 secretion promoted resistance of breast cancer cells to chemotherapy; whether or not the RGD motif in IGFBP-2 is responsible for these actions is still unclear and needs further investigation.

Our results have potential implications for cancer patients who suffer from type 2 diabetes, implying that rigorous control of their glucose levels might increase their responsiveness to chemotherapy. Supporting this is evidence suggesting that sufficient control of hyperglycaemia during chemotherapy could improve outcome of treatments in patients with haematological and solid tumours [31]. In addition, accumulating epidemiological and laboratory studies have reported that the use of antidiabetic therapy may improve responsiveness of cancer cells, enhanced effectiveness to chemotherapy, and reduce recurrence. Agents such as metformin have increasingly become of interest in cancer treatment; though the mechanisms of how these agents act and their anticancer effects are still being investigated and defined. Adding metformin to adjuvant breast chemotherapy in nondiabetic individuals reduced glucose levels, improved sensitivity to insulin and appeared to reduce subsequent metastasis [32]. In mice, reduction of glucose levels by fasting prior to chemotherapy improved the outcome of therapy, lowered toxicity and reduced IGF-I levels [33]. Recently, our group has shown that metformin could restore the sensitivity of prostate cancer cells to docetaxel. Treating prostate cancer cells with docetaxel in hyperglycaemic conditions reduced cell death and induced resistance. This resistance against docetaxel was negated when the cells were treated with metformin and this was mediated by a reduction in the levels of IGFBP-2 [34].

As the tumour grows, the tumour cells can frequently outgrow the blood supply resulting in the cells being exposed to hypoxia. The results presented here indicate that in euglycaemic conditions exposure to hypoxia does not affect the response of breast cancer cells to chemotherapy. In contrast, in hyperglycaemic conditions hypoxia can completely negate the hyperglycaemia-induced resistance to chemotherapy. Although in many solid tumours cell metabolism is altered with an increase in glycolytic flux and a reduction in flux through the TCA cycle with reduced oxidative phosphorylation, often termed the Warburg effect. This effect is used clinically in an imaging technique, PET, to localise solid tumours. In the prostate cell metabolism is atypical and PET has proven of limited use for prostate cancer. Our results indicate that the effects of hyperglycaemia on chemosensitivity are probably dependent on oxidative phosphorylation. Our study has also shown that hypoxia reduces IGFBP-2 and FASN. Since FASN and IGFBP-2 confer resistance to chemotherapy in high glucose, reducing FASN and IGFBP-2 abundance negated the effect of hyperglycaemia under hypoxia. Hyperglycaemia has been shown to impair the expression of a number of HIF family members. HIF-Iα repression by hyperglycaemia contributes to many complications in diabetes [35]. In non-small cell lung cancers, the ATP-dependent efflux of doxorubicin by P-glycoprotein (P-gp) has been reported to be one important mechanism for resistance. Increased expression of P-gp was associated with increased doxorubicin efflux and induced resistance to doxorubicin. Hypoxia negated this P-gp mediated resistance to doxorubicin via HIF-Iα signalling [36]. Since IGFBP-2 expression and secretion is increased in high glucose and IGFBP-2 has a role in inducing resistance to chemotherapy in breast cancer cells, this may suggest that IGFBP-2 might have an effect on P-gp, which would need to be investigated. In conclusion, the response of breast cancer cells to chemotherapy is dependent on the conditions of the tumour microenvironment. High glucose increased the abundance and expression of IGFBP-2 in breast cancer cells that led to reduced sensitivity to chemotherapy. The hyperglycaemia-induced chemo-resistance was negated by hypoxia and this was consistent with a reduction in IGFBP-2 levels.

## MATERIALS AND METHODS

Compounds were purchased from Sigma-Aldrich, Dorset, UK unless otherwise stated.

### Cell maintenance and treatment

Breast cancer cells MCF-7 and T47D, purchased from ATCC (Molsheim, France) were cultured and maintained in 4.5g glucose Dulbecco’s modified Eagle’s medium (DMEM) supplemented with 10% fetal bovine serum (FBS) and 2mM L-glutamine. Upon 80% confluence, cells were detached from culture flasks using 10% trypsin. The cells were serum starved for 24h in serum-free DMEM in normal (5mM), well-controlled diabetic (9mM), or uncontrolled severe hyperglycaemic (25mM) levels of glucose. Depending on the context of the experiment, the cells were treated with human recombinant IGFBP-2 (GroPep, Thebarton, Australia), 17β-estradiol-water soluble, 17β-estradiol 6-(o-carboxy-methyl) 31.20 mole steroid/mole BSA oxime:BSA, or doxorubicin hydrochloride. Hypoxia was induced chemically using 500μM CoCl_2_ or by the use of a hypoxia chamber set to 2% oxygen (O_2_) pressure.

### Transfection with siRNA

IGFBP-2 siRNA was used as described previously [11]. The transfection kit was purchased from Synvolux Therapeutics (Leiden, The Netherlands) and transfection was conducted following the manufacturer’s instructions.

### Trypan blue dye exclusion assay

Viable and dead cells were determined by trypan blue dye exclusion assay as described previously [14]. Dead cells were defined as those stained dark blue whereas live cells are bright white.

### Protein analysis

Western immunoblotting was conducted to monitor changes in protein abundance, and band intensities were determined by ImageLab software as described by the manufacturer BioRad, Hertfordshire, UK. The membranes were probed with anti-FASN (mouse, 1:2000) and anti-HIF-Iα (mouse, 1:500), purchased from BD Biosciences Oxford, UK, anti-ERα (mouse, 1:750) and anti-IGFBP-2 (goat, 1:1000) from Santa Cruz Heidelberg, Germany, anti-Akt (rabbit, 1:1000) and anti-p-Akt (rabbit, 1:1000) purchased from Cell Signaling Hertfordshire, UK, and anti-α-tubulin (mouse, 1:5000) purchased from Merck Millipore Hertfordshire, UK. Depending on the species of the primary antibody, the blots were incubated in horseradish peroxidase-linked anti-mouse/rabbit/goat antibody (1:2000) purchased from Merck Millipore Hertfordshire, UK.

### Quantitative real time polymerase chain reaction (qRT-PCR)

Breast cancer cells were exposed to different glucose levels (5mM, 9mM and 25mM glucose) for 24h prior to extraction of RNA as outlined previously [37]. 18S was used as a housekeeping gene.

### Enzyme-linked immunosorbent assay (ELISA)

ELISA was used to estimate the concentration of secreted IGFBP-2 expressed in ng/ml as performed previously [11]. ELISA was performed following the manufacturer’s instructions (DuoSet ELISA kit Human IGFBP-2, Abingdon, UK).

### Statistical analysis

Data in this study represent the mean±SEM of three independent experiments. The standard curve of ELISA outputs followed a four parameter logistic (4-PL) curve fit. For multiple comparisons, one-way ANOVA post hoc LSD test was used to compare treated samples with controls using IBM SPSS Statistics 21 software. Differences between samples were considered significant at *P<0.05, **P<0.01, and ***P<0.001.

## SUPPLEMENTARY MATERIALS FIGURES



## Abbreviations

CoCl_2_: cobalt chloride
ERα: estrogen receptor α
FASN: fatty acid synthase
HIF-I: hypoxia-inducible factor-I
IGFBP-2: insulin-like growth factor binding protein-2
