# Supplementary material for: Hypoxia negates hyperglycaemia-induced chemo-resistance in breast cancer cells: the role of insulin-like growth factor binding protein 2
Source: Oncotarget. 2017 Aug 16;8(43):74635–48. doi: 10.18632/oncotarget.20287 (PMC5650368; doi:10.18632/oncotarget.20287)
Supplement: Supplementary file 1 [file oncotarget-08-74635-s001.pdf]

Hypoxia negates hyperglycaemia-induced chemo-resistance in breast cancer cells: the role of insulin-like growth factor binding protein 2

SUPPLEMENTARY MATERIALS

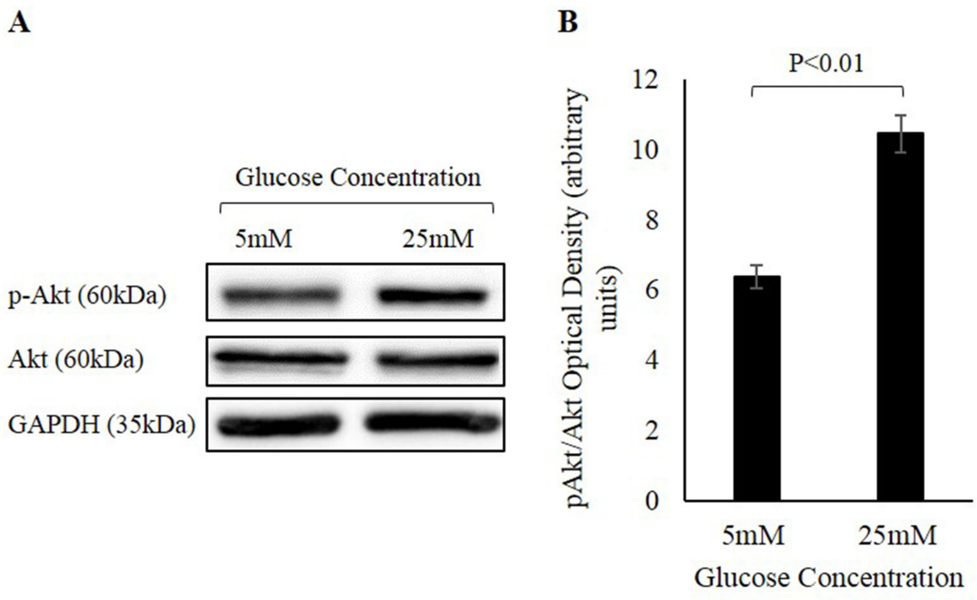

Supplementary Figure 1: The effect of different glucose levels on Akt. **(A)** MCF-7 cells were treated in 5mM and 25mM glucose and the levels of Akt phosphorylation was identified by Western immunoblotting. **(B)** The densitometry measurements of pAkt/Akt in different glucose levels. The graphs represent the mean±SEM of three independent repeats.

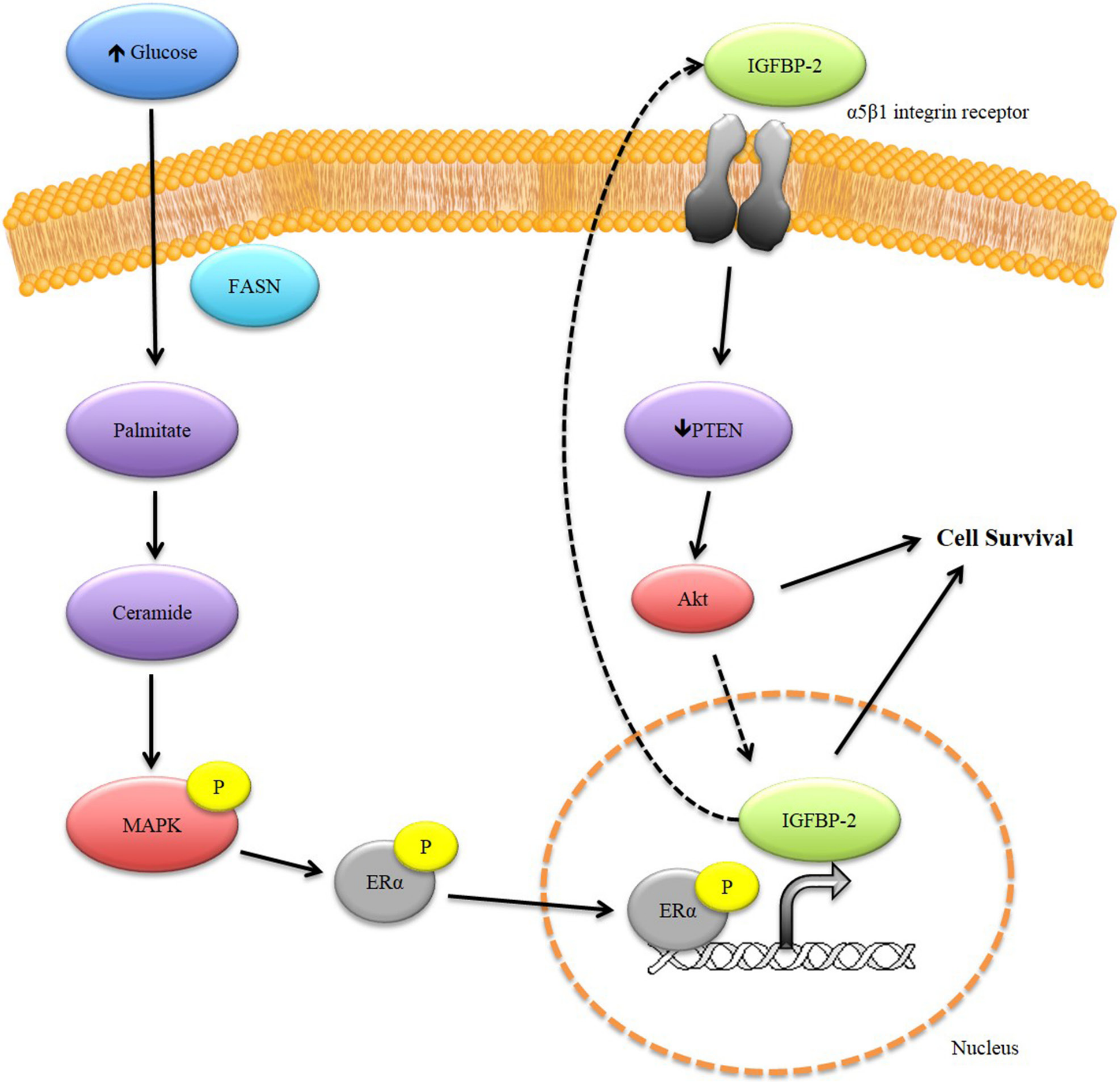

Supplementary Figure 2: Illustration of hypothesised pathway by which IGFBP-2 affects the response of breast cancer cells to chemotherapy in high levels of glucose. Lipid metabolism is regulated by FASN, which converts glucose to palmitate. This conversion triggers subsequent signalling cascades involving ERα being phosphorylated and translocated to the nucleus where it acts as a transcriptionally promotes the expression of IGFBP-2. IGFBP-2 being secreted outside the cells acts in a positive feedback loop promoting more induction of IGFBP-2.
